# The Impact of Antimicrobial Stewardship Training on Calf Producers’ Knowledge, Treatment Behaviors and Quantified Antimicrobial Use

**DOI:** 10.3390/microorganisms10081525

**Published:** 2022-07-27

**Authors:** Jessica Pempek, Martey Masterson, Rafael Portillo-Gonzalez, Kate Creutzinger, Ting-Yu Cheng, Greg Habing

**Affiliations:** 1 Department of Animal Sciences, College of Food Agriculture and Environmental Sciences, The Ohio State University, Columbus, OH 43210, USA; pempek.4@osu.edu; 2 Department of Veterinary Preventive Medicine, College of Veterinary Medicine, The Ohio State University, Columbus, OH 43210, USA; masterson.2@osu.edu (M.M.); portillo-gonzalez.1@osu.edu (R.P.-G.); cheng.1784@osu.edu (T.-Y.C.); 3 Department of Animal and Food Science, University of Wisconsin-River Falls, River Falls, WI 54022, USA; katherine.creutzinger@uwrf.edu

**Keywords:** antimicrobial stewardship, antimicrobial use, dairy calves, antimicrobial resistance, veal, epidemiology

## Abstract

The judicious use of antimicrobials on farms is necessary to mitigate the development of antimicrobial-resistant pathogens that compromise human and animal health. On livestock farms, veterinarians prescribe and dispense antimicrobials, but producers use rapid judgements of disease severity to make routine decisions on the initiation of empirical antimicrobial therapy. Therefore, the knowledge and skills required to accurately diagnose treatable bacterial infections is necessary for optimal antimicrobial stewardship. Veal calves often undergo stressors and environmental exposures that increase calves’ risk of bacterial infections, and antimicrobials are sometimes necessary to ensure their health. The objective of this trial was to measure the impact of antimicrobial stewardship training on calf producers’ knowledge of antimicrobial stewardship, accuracy of identifying calves for treatment, and quantified antimicrobial use. Eight farms were evenly allocated into either intervention or control groups. Training resulted in both higher scores on assessments and higher sensitivity for detecting cases that required antimicrobial therapy relative to a veterinarian. Importantly, there was a 50% reduction in the antimicrobial dosing rate among intervention farms relative to control farms. Antimicrobial stewardship training among calf producers was effective at changing producers’ behaviors and reducing antimicrobial use.

## 1. Introduction

Medically important antimicrobials are routinely used for the treatment of livestock diseases, in order to mitigate the impact of bacterial infections on the animals’ health and welfare. However, the use of antimicrobials on farms contributes to the development of antimicrobial-resistant (AMR) pathogens that affect humans and animals. Therefore, sustainable livestock production will require improvements in the stewardship of antimicrobials for the sake of protecting human health. In the United States, the use of medically important antimicrobials requires a veterinary client patient relationship [1], and veterinarians have responsibilities for the oversight of antimicrobial use (AMU), including the creation of farm protocols. Nonetheless, livestock producers are responsible for making routine decisions on the initiation of therapy in the absence of a veterinarian [2]. Therefore, optimal antimicrobial stewardship requires producers with knowledge and skills to accurately identify animals that require antimicrobial therapy.

Dairy beef and veal calf production systems are under-recognized and important sources of AMR pathogens. The neonatal microflora in any young calf has high levels of AMR bacteria, but male dairy calves are particularly susceptible [3], as they often receive discrepantly worse care on the dairy farm compared to their heifer counterparts, including suboptimal colostrum practices [4,5]. Male dairy calves are typically sold in the first week of life, aggregated at livestock auctions and buying stations, and are transported to dairy beef or veal calf production systems throughout the Midwest [4]. The combination of immune deficiency and high-risk environmental exposures makes calves an ideal vehicle for the dissemination of AMR pathogens and makes them susceptible to bacterial infections after their arrival to growing facilities. Approximately 75% and 90% of fecal samples from 3–4-month-old veal calves had *E. coli* that were resistant to the critically important antimicrobials ciprofloxacin and ceftiofur, respectively, which led to relatively high levels of AMR pathogens on chilled carcasses [6]. Additionally, a recent National Antimicrobial Resistance Monitoring System (NARMS) survey (May 2021) documented high levels of resistance in retail veal relative to other retail meats [7].

In order to mitigate the impacts of AMU, antimicrobials should only be used when necessary for animal health [1]. Ideally, producers would identify all calves requiring antimicrobial therapy to maintain optimal welfare (i.e., high diagnostic sensitivity), and only treat those calves that genuinely require antimicrobial therapy (i.e., high specificity). Still, 40% of producers who were presented with vignettes of mild cases of gastrointestinal disease (i.e., no systemic signs) selected antimicrobials as a typical therapy, suggesting that there are opportunities to better target AMU without negative impacts on animal health [2].

Improvements in antimicrobial stewardship will require evidence-based interventions and changes in producer treatment behaviors. The required behavioral changes are difficult to achieve, and improved knowledge and/or training is necessary but sometimes insufficient since behavior is additionally influenced by external and psycho-social factors. Therefore, though knowledge and skill deficits related to antimicrobial stewardship exist among farm personnel, it is not known if providing training will necessarily reduce antimicrobial use. Integrated animal production systems offer the opportunity to allocate interventions to farms to estimate farm-level effects on stewardship outcomes. Given the necessity of passing knowledge and skills onto producers so that they can accurately identify animals requiring antimicrobial therapy, we conducted a trial to determine the ability of a training program focused on producer-oriented antimicrobial stewardship education, in order to improve treatment accuracy and reduce the quantity of antimicrobials used within an integrated veal production system. We hypothesized that producer-focused education would improve knowledge, lead to more accurate identification of calves in need of treatment, and result in less quantified antimicrobial use.

## 2. Materials and Methods

### 2.1. Study Design

In order to test our hypothesis, we conducted a field trial. Eight farms from a single veal calf production system in the midwestern U.S. were selected based on their proximity to the Ohio State University Columbus campus, and their willingness to participate in the data collection. Four of the farms were assigned to receive the training intervention, based on their willingness and availability to participate in the didactic and hands-on training from the investigative team. Five farms within each treatment group were originally planned to provide sufficient power (1 − β = 0.80) to find a significant (α = 0.05) change within each study group between two time points (10 paired observations), assuming a mean antimicrobial treatment incidence of 0.3 and 0.15 doses/calf-day for time points 1 and 2, respectively, and a correlation between within-herd observations of 0.5. However, only eight farms were receiving cohorts of calves and enrolled.

**Figure 1:**
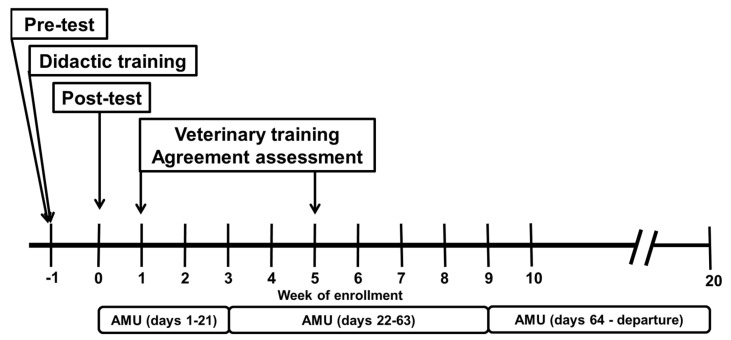
Timing of activities on a project designed to improve antimicrobial stewardship on veal production farms. Changes in knowledge were assessed among producers in the intervention group using a pre- and post-assessment that were administered immediately before and 1 week following the didactic training, respectively. Calf producers received hands-on training at 1 and 5 weeks of the study, and their agreement with the veterinarian on identification of calves for treatment was assessed at the same time points. The quantity of antimicrobials used was measured across three consecutive periods following the arrival of a calf cohort (days 1–21, 22–63, 64–departure).

Within this veal production system, barns remain empty for approximately 3 weeks after the departure of a cohort of calves for harvest and before the arrival of a new calf cohort. The didactic training for each enrolled farm was timed to begin approximately one week prior to the arrival of the new cohort of calves (Figure 1). Producers’ knowledge was measured on both intervention and control farms prior to the intervention, and changes in knowledge resulting from the training were measured among producers on intervention farms 1 week following the didactic component of the intervention. Data collection on quantified antimicrobial use began on the day of calf arrival and was assessed throughout the growing period. Treatment accuracy was measured on intervention and control farms at 1 and 5 weeks after the arrival of a new calf cohort to capture periods of highest risk for gastrointestinal and respiratory disease, respectively.

Eight farms and a total of ten producers were enrolled. Each of the enrolled producers was responsible for identifying sick calves and making decisions on the initiation of individual and group antimicrobial treatments. Two individuals from two of the intervention farms completed the training, and one individual from the remaining two intervention farms completed the training. Among the control farms, one individual from each of four farms was enrolled and completed the knowledge assessment. One farm in the control group declined ongoing participation three weeks after enrollment, hence antimicrobial use data from this farm were included for the first rearing period only (days 1–21).

### 2.2. Setting

Each of the eight farms used an all-in-all-out animal flow, where barns were filled with calves over a 1- to 3-day period; the mean cohort size of the farms was 212.2 (standard deviation = 67.7) calves, with a range of 120 to 315 calves per cohort. Arriving calves were generally purchased from auctions or from other calf dealers, aggregated by calf dealers at buying stations, and arrived at the veal farms by approximately 1 week of age. On arrival to the farms, calves were housed in individual stalls (approximately 2.13 m × 0.61 m) with wooden (6 farms) or metal (2 farms; Tenderfoot^®^, Tandem Products, Inc., Minneapolis, MN, USA) slatted flooring. Removable metal dividers with partitions for group separation were used to provide visual access among calves. Pens were changed to accommodate group housing (2 to 9 calves per group) in the eighth week. Calves were fed milk replacer (MR; 22% protein, 18% fat) twice via buckets or troughs, every day at approximately 05:00 and 16:00. Each feeding began with 1.47 kg of MR containing 220 g of MR powder on day 1 of the study, and was then gradually increased to 2.95 kg of MR prepared by reconstituting 454 g powder on day 21 of study. Drinking water was offered ad libitum via bucket or trough while in individual housing, and via metal nipple in group housing. A starter grain mixture was offered to all calves ad libitum throughout the growing period.

### 2.3. The Intervention

The intervention comprised three components: didactic presentations, calf-side training and veterinarian feedback. The 2-hour in-person didactic training, conducted by JP, RPG and GH, included three modules, each with four learning objectives (Table 1). The first through third modules focused on antimicrobials and antimicrobial resistance, calf health assessments and decision tree treatment protocols, respectively, as detailed in the learning objectives within Table 1. Ultimately, the overall goal was to improve the adherence of producers to existing veterinary-written protocols, and to have them select the appropriate individual calf treatment strategy based on these existing protocols. Directly following the presentations, a member of the team reinforced the didactic content by working directly with the producer on individual calf health assessments, focusing on semi-objective scoring assessments for navel infection, diarrhea, dehydration and respiratory disease that were incorporated into veterinary-written decision tree protocols. The producers worked directly with their veterinarian during the measurements of treatment accuracy (see description below). During this process, the veterinarian identified instances where the producer recorded different antibiotic use decisions relative to the treatment protocol, and these discrepancies were discussed in an unstructured fashion. Thus, these measurements were useful to reinforce didactic content, to clarify points of confusion, and to identify specific instances where producer decisions disagreed with the veterinary-written protocol. Additionally, calf health assessments were reinforced within unstructured discussions during subsequent visits at 1, 3, 5, 9, 14, 20 and 23 weeks. All decision tree protocols and didactic resources are available at (https://u.osu.edu/oneherdlab/teaching/resources-for-calf-producers/, accessed on 30 June 2022).

### 2.4. Knowledge Assessment

A 31-item assessment was developed to assess producer knowledge of calf health and the appropriate use of antimicrobials. General topics for the items included antimicrobials, antimicrobial resistance, calf-health assessments, and vignette-based scenarios to assess common treatment practices [2]. The same questionnaire was given to the enrolled individuals in both the intervention and control groups. For individuals in the intervention group, the questionnaire was given just prior to the beginning of the training, and the same questionnaire was re-administered approximately one week following the training. Questionnaire items included multiple-choice with one correct answer, multiple-choice with multiple correct answers, true or false, and fill in the blank. Within the 31 items, there were a total of 69 possible correct responses. The number of correct responses (out of 69) were tabulated for each producer for the pre- and post-intervention questionnaire.

### 2.5. Assessment of Treatment Accuracy

In order to assess the adherence of the producers to the veterinary-written antimicrobial use protocols, each enrolled producer (*n* = 10) and the veterinarian of record (MM) independently assessed the 75 calves on two occasions, approximately 1 week and 5 weeks following calf arrival; these times coincided with the typical peak incidence of gastrointestinal and respiratory disease within the production system, respectively. The veterinarian (MM) assessed the 75 calves and recorded if antimicrobial therapy should be administered according to the written protocol. Each producers’ diagnostic sensitivity was calculated as the proportion of calves requiring antimicrobial therapy (as determined by the veterinarian) that were correctly identified as requiring antimicrobial therapy by the producer. Similarly, the diagnostic specificity was defined as the proportion of calves not requiring antimicrobial therapy where the producer did not select antimicrobial treatment. On intervention farms, the veterinarian and producer discussed decision discrepancies following the objective assessments, in order to additionally improve producer calf health assessment skills. On control farms, the veterinarian assessment of the 75 calves was compared to the treatments recorded by the producer on the same day. 

### 2.6. Quantification of Antimicrobial Use

Quantification of antimicrobial use on the eight enrolled farms has been previously described (Cheng et al., 2022). Briefly, antimicrobial use was quantified separately for group and individual administrations of antimicrobial agents by calculating treatment incidence (TI) as a function of antimicrobial agent daily doses, animal weight and rearing periods. The number of antimicrobial doses was calculated by dividing the mass of antimicrobials (measured through the collection of empty drug containers) by the daily dose extracted from farm protocols (i.e., used daily dose, UDD) for the antimicrobial and the estimated mean calf weight at three different periods during the growing period (Figure 1). In this case, farm protocol dose definitions were used in place of defined doses to more accurately reflect changes in the numbers of doses administered on the farm. The numbers of doses used were converted into a treatment incidence (TI-100) by dividing by the total number of calf-days during the measurement period (Equation (1)). The TI-100 can be interpreted as the mean number of doses used for each calf per 100 days. Separate estimates of AMU were made for days 1–21, 22–63 and 64–departure. The first two time periods capture the time periods with the highest risk for gastrointestinal disease and respiratory disease, respectively.

(1) $$\mathrm{TI}100=\frac{\mathrm{total}\;\mathrm{mass}\;\mathrm{of}\;\mathrm{used}\;\mathrm{antimicrobial}\;(\mathrm{mg})}{\mathrm{UDD}\;(\mathrm{mg/kg})\;\times \;\mathrm{calf}\;\mathrm{weight}\;(\mathrm{kg})\;\times \;\mathrm{calf-days}\;\mathrm{at}\;\mathrm{risk}}\times 100$$

### 2.7. Statistical Analysis

Producer knowledge was measured as the proportion of correct responses on the questionnaire (no. of correct responses/no. of possible correct responses). We used a generalized linear mixed model (PROC GLIMMIX; SAS Version 9.4, Cary, NC, USA) to compare changes in the proportion of correct questions and responses on the assessment before and after the training, where the binomially distributed dependent variable was the proportion of questions or responses (i.e., events/trials) answered correctly. The model included time point (pre vs. post) as a fixed effect, and producer as a random intercept.

The proportions for producer diagnostic sensitivity and specificity were descriptively compared between intervention and control herds, and between the first and second visits. A modified Poisson regression model (PROC GENMOD; SAS Version 9.4, Cary, NC, USA), which has the ability to directly estimate risk ratios [8], was constructed in order to test the hypothesis that calves requiring antimicrobial therapy (as assessed by the veterinarian) had a higher risk of treatment on intervention farms (i.e., higher producer sensitivity). A second similar model was constructed to compare the risk of treatment among calves assessed by the veterinarian as not requiring antimicrobial therapy (i.e., producer specificity). In each of the models, a repeated effect for farm was included, and assessment number (pre or post) and treatment group (intervention or control) were included as fixed effects. A statistical interaction between visit and treatment group was offered to the model. The risk ratio and associated confidence intervals for treatment by study group and visit was estimated from each model.

The descriptive and statistical analyses of quantified antimicrobial use were conducted using the fundamental features of R (R Foundation for Statistical Computing, Version 4.0.4, Vienna, Austria). In order to determine the effect of the intervention on antimicrobial use, the treatment incidence of all antimicrobial uses was compared between intervention and control farms for rearing periods 1 to 3 (days 1–21, 22–63 and 64–departure) using generalized linear mixed regression models based on the Poisson distribution and natural logarithm link function. Prior to the model’s construction, the numbers of doses were rounded up to the nearest whole numbers to accommodate the Poisson distribution. Two separate models for the dosing rate at individual and group treatment levels were constructed including the rounded number of doses (response) with calf-day as the offset, the intervention status, rearing periods (periods 1 to 3), and their interaction (fixed effects) and farm (random effect). Least square means and pairwise comparisons among fixed effects were estimated using Bonferroni-adjusted post hoc analysis. Within each treatment level, the treatment incidence rate ratio (IRR) in intervention relative to control groups was estimated for each rearing period, along with adjusted *p*-values and 95% confidence intervals. The same modeling approach without the rearing period and interaction fixed effects were used to evaluate the effect of the intervention on the total amount of antimicrobials used throughout the entire rearing period. Lastly, least square means and confidence intervals for the carcass weights were calculated by constructing a generalized linear mixed model, where weight was the outcome and barn was included as a random effect.

## 3. Results

### 3.1. Changes in Knowledge

Among the 10 individuals from intervention and control farms taking the questionnaire without (control farms) or prior (intervention farms) to receiving the intervention, the mean proportion of correct responses was 0.81, 0.73 and 0.77 on items linked to content in modules 1 (Antibiotic Use and Resistance), 2 (Calf Health Assessments) and 3 (Decision Tree Protocols), respectively (Table 2). The pre-test revealed important knowledge gaps; for example, only half the producers (5/10) correctly identified the antimicrobials from a list of medications commonly used on the farms. Two of the ten producers correctly indicated the rectal temperature which constituted a fever based on the written treatment protocol (i.e., ≥102.5 F or ≥103.0 F). Eight of the ten respondents correctly indicated that antimicrobials are used to treat bacterial rather than viral or fungal infections. Four of the ten producers indicated that they might treat calf diarrhea (without any other systemic signs) with antimicrobials. 

In total, producers in the intervention group selected significantly more correct responses on the post-test compared to their own results on the pre-test (*p* = 0.05). Among the six producers in the intervention group, the mean proportion of correct responses increased modestly from 0.74 (range: 0.58–0.83) to 0.81 (range: 0.69–0.90) before and after the didactic intervention, respectively. Overall, the number of correct responses increased by a mean of six (range: 0–16) among the total of 69 possible responses. Scores improved numerically for all modules, but the percentage increase in the proportion of responses correct was 16% for module 2 (Health Assessments) after the intervention, compared to 3% and 10% for modules 1 and 2, respectively.

### 3.2. Differences in Treatment Accuracy among Intervention and Control Farms

**Figure 2:**
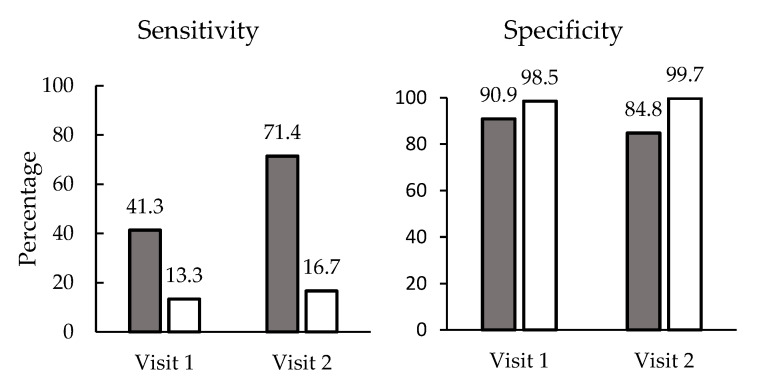
Diagnostic sensitivity and specificity of calf producers for identifying calves requiring antimicrobial therapy, relative to the veterinarian of record for the farms. Individuals (*n* = 6) in the intervention group (closed bars) received training on disease identification for navel disease, diarrhea and pneumonia. Individuals (*n* = 4) in the control group (open bars) did not receive the training. The assessments were made at approximately 1 week and 5 weeks following the arrival of a cohort of calves, in order to capture time periods of highest risk. Individuals in the intervention group had significantly higher sensitivity and lower specificity relative to the veterinarian.

Among two visits at approximately 1 week and 5 weeks after calf arrival on the eight enrolled farms, the veterinarian identified 209 calves with conditions where antimicrobials were designated as an appropriate treatment, as indicated by the veterinary written protocol, including 146 calves used to assess 6 individuals on the intervention herds, and 63 calves used to assess the 4 enrolled individuals on control farms. Calf producers within intervention herds had a significantly higher sensitivity than control herds (*p* = 0.002). Producers on intervention farms correctly identified 50% (73/146) of the cases, compared to 14.3% (9/63) on control farms (Figure 2). Calves deemed by the veterinarian to require antimicrobial therapy on the intervention farms had 3.53 times the risk of treatment (95% CI: 1.77–7.07), compared to those on the control farms. Across the four intervention herds, the calf producer sensitivity ranged from 30% to 53%, and on the four control farms, the sensitivity ranged from 5.9% to 26.1%. Producers were less likely (*p* < 0.001) to correctly identify cases to be treated on visit one (32.9%, 49/149 cases) relative to visit two (55.0%, 33/60 cases), when gastrointestinal disease and respiratory disease, respectively, are generally more common.

Similarly, the veterinarian at the same visit assessed 550 and 664 calves in the control and intervention herds, respectively, that were deemed to not require antimicrobial therapy. The specificity for antimicrobial treatment decisions was lower on intervention farms (87.6%, 665/758 cases) compared to that on control farms (99.1%, 550/555 cases) (Figure 2). For intervention farms, the specificity ranged from 84.3% to 90.6%, and ranged from 97.8% to 100% on control farms. Calf producer specificities of 94.1% (579/615) and 91.0% (635/698) on the first and second visit were not different for either treatment group (*p* = 0.49).

### 3.3. Differences in Quantified Antimicrobial Use

**Figure 3:**
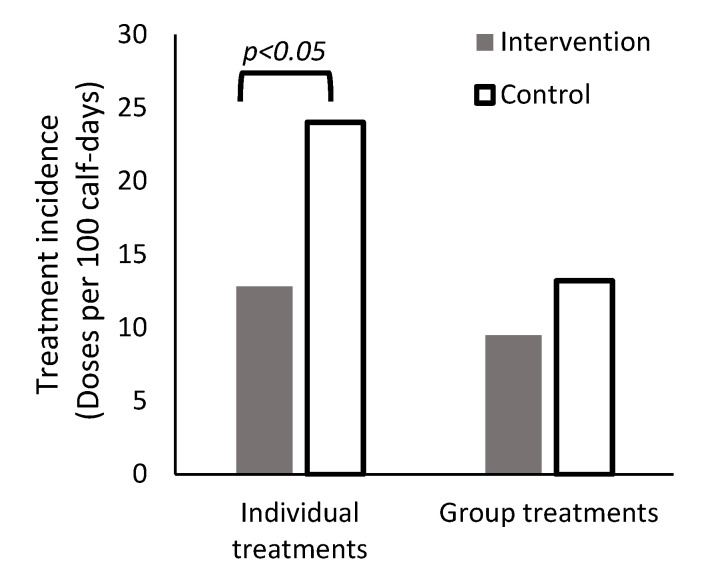
Quantification of antimicrobial use on veal farms randomized to receive an antimicrobial stewardship intervention (closed bars) and control farms. Dosing with individual antimicrobial agents was reduced by 50% among intervention farms.

There were important and significant reductions in the dosing rate for individually administered antimicrobials across time periods on intervention farms relative to control farms (Table 3). During the entire rearing period, intervention farms used a mean of 12.8 doses/100 calf-days for individually administered antimicrobials compared to 24.0 doses/100 calf-days on control farms, which is an estimated 50% decrease (IRR = 0.50, 95% CI: 0.30–0.84) (Figure 3). There was a significant interaction between the intervention status and rearing periods for all models, indicating that the effect of the intervention was different across time periods (interaction term F test, *p* < 0.05). A numerically larger (but not statistically different) TI100 for individually administered antimicrobials was observed in the first rearing period (IRR = 1.14, 95% CI: 0.64–2.02); however, the TI100 was numerically lower in period 2 (28% decrease; IRR = 0.72, 95% CI: 0.40–1.27), and significantly lower in period 3 (47% decrease; IRR = 0.53, 95% CI: 0.30–0.95) (Table 3).

Over the entire period, farms in the intervention and control groups used 22.3 ± 9.6 (mean ± standard deviation) and 37.2 ± 10.4 group-administered doses per 100 calf-days, respectively. The treatment incidence was not statistically different between farm groups for the entire rearing period (IRR = 0.73, 95% CI: 0.76–2.19) and the first period (IRR = 1.29, 95% CI: 0.75–2.12); however, the TI100 was significantly lower for the second period (IRR = 0.59, 95% CI: 0.35, 1.00) and the third period (42% decrease; IRR = 0.58, 95% CI: 0.34, 0.99). 

The TI100 for specific antimicrobials are described in Table 3. Briefly, among 15 different antimicrobials used among the enrolled farms, the most administered antimicrobials were chlortetracycline HCL, penicillin G procaine and amoxicillin. All of the antimicrobials are classified as either critically important (*n* = 8), highly important (*n* = 6) or important (*n* = 1) by the World Health Organization. Among the eight critically important antimicrobials (CIA), four (three macrolides and a 3^rd^ generation cephalosporin) are classified as highest priority critically important (HP-CIA), including tulathromycin, tylosin, tildipirosin and ceftiofur [9]. Seventy-nine percent (11/14) of the individually administered antimicrobials had a TI100 numerically lower in the intervention group relative to the control group. Among the HP-CIA antimicrobials, the use of ceftiofur and tylosin was higher in the intervention group, while the use of tulathromycin and tildipirosin was lower in the intervention group (Table 3). The largest difference in the TI100 between intervention and control group was among the beta-lactam antimicrobials (37% lower), lincosamides (67% lower) and aminoglycosides (56% lower).

### 3.4. Production Information among Calf Cohorts

Mortality, carcass weight and the proportion of carcasses condemned were similar between intervention and control farms. The mean carcass weight ranged from 260.3 to 289.4 among the enrolled barns and was numerically higher among the intervention barns. For intervention and control barns, the adjusted mean and 95% confidence limits for carcass weight were 275.7 (261.8–289.6) and 274.1 (258.1–290.1), respectively. Additionally, 0.68% (4/562) of carcasses were condemned on intervention farms and 0.71% (5/521) of carcasses were condemned on control farms. Lastly, calf mortality was numerically lower on intervention farms. Twelve percent (78/640) of calves died on farms prior to slaughter for intervention farms, compared to 13% (79/600) calf deaths for control farms. 

## 4. Discussion

This trial determined the impact of antimicrobial stewardship training materials on the knowledge, treatment behaviors and quantified antimicrobial use of veal calf producers. Evidence-based approaches to antimicrobial stewardship required appropriate testing of interventions in order to reduce the use of medically important antimicrobials on livestock production farms. For instance, U.S. hospitals have demonstrated reductions in defined daily doses through the implementation of antimicrobial stewardship programs [10,11]. Similarly, the integrated nature of veal calf production allowed for farm-level allocation to an intervention or control group, enabling estimation of farm-level effects. Additionally, the close collaboration and work from the veterinarian of record (MM) was essential for successful implementation and effective training. Producers use rapid judgments of disease severity to make decisions on when to begin a course of antimicrobial therapy, and training is necessary for judicious use. Relatively minor alterations to calf producer case definitions of disease could have important impacts on antimicrobial dosing rates. Importantly, the goal of this study was not to shift the types of antimicrobials used, but rather to improve the ability of calf producers to correctly identify animals that required treatment.

### 4.1. Improvements in Knowledge of Antimicrobial Stewardship

Among the six individuals from the four intervention farms, there were statistically significant improvements in knowledge on antimicrobial stewardship. Still, the measured improvements in assessment scores were modest, and additional or reinforced training is likely to be important for more substantial and sustained knowledge transfer. In part, the modest improvement in knowledge may be because the post-test was administered one week following, rather than immediately after, the didactic training. Furthermore, the assessments did not capture changes in knowledge or treatment behaviors resulting from the case discussion and calf-side demonstration, which occurred after the post-test (Figure 1). The largest improvement in knowledge occurred in the second module (Calf Health Assessments), which is an important module for improvements in the diagnostic accuracy and dosing rate among the producers.

### 4.2. Changes in Diagnostic Sensitivity and Specificity among Calf Personnel

The training appeared to improve calf producer sensitivity for detecting cases that required antimicrobial therapy; however, the specificity of producers was lower on intervention farms relative to control farms. The apparent tradeoff in sensitivity and specificity when refining case definitions over a spectrum of disease severity presentations is somewhat intuitive, as adjustments to a cut-off to improve the sensitivity of diagnostic tests often have opposing effects on the test specificity [12]. In this case, enhanced disease awareness among producers and prioritization of complete identification of disease cases for improved calf health can manifest as higher diagnostic sensitivity with a lower specificity. This is an important consideration for future stewardship efforts. The increase in sensitivity and decrease in specificity among intervention farms would be expected to increase the dosing rate, and so these results contradict the significant reductions in antimicrobial dosing (TI100) for individual disease in the second and third periods of the growing period (Table 3). The reasons for the apparent contradiction are not known for certain, but the two days of assessments at approximately 1 and 5 weeks may not adequately represent treatment behaviors over the ~150 day growing period. Additionally, feedback received from the visiting veterinarian following the assessments may have resulted in important changes in treatment decisions that were not captured by the assessments. Calf producers were assessed with the knowledge that they would be compared with the visiting veterinarian, and the decisions may have been different for other days during the growing period for the cohort of calves. Future studies should incorporate more frequent treatment accuracy assessments when evaluating antimicrobial stewardship interventions.

### 4.3. Reduction in Quantified Antimicrobial Use

The dosing rate for individually administered antimicrobials on farms that had received the training was half of the dosing rate administered on control farms. The educational intervention was primarily targeted towards improving the accuracy of individual antimicrobial treatments, and improvements in treatment accuracy are particularly important for reducing the use of CIA that are administered individually (e.g., ceftiofur, tulathromycin). Antimicrobial use in the intervention group was significantly lower in the models for the dosing rate for the entire period, numerically higher in the models for the first period, numerically lower for the second period and significantly lower for the third period. Most of the AMU dosing occurred in the first period, and additional efforts should be targeted towards AMU during this time frame, including appropriately stringent treatment decisions for navel and gastrointestinal conditions. Creation of these decision tree protocols are limited by the lack of research on the effects of antimicrobial therapy for routine cases of gastrointestinal disease. Regardless, more substantial differences in quantified AMU may have manifested in the second and third periods due to ongoing veterinarian feedback at the assessments that occurred during the first and fifth weeks. Additionally, the decision tree protocols used to inform the treatment of respiratory disease (primarily second period) are well developed, previously validated [13], and potentially more specific than the gastrointestinal disease and navel disease protocols used on these farms, primarily in the second period of the grow.

The outcome measurement chosen for this study was the treatment incidence rate for antimicrobial dosing per 100 calf-days. Other studies on antimicrobial use monitoring programs have used similar metrics [14,15]. In our case, the doses are defined by the farm protocol (i.e., used daily dose), rather than by the labeled dose or dose definitions available through the European Medicines Agency (EMA) (EMA, 2016). Based on a prior characterization of AMU on these herds, the UDD underestimated AMU relative to dose definitions based on the label for AMU [16]. Still, the UDD is more apt to detect changes in the dosing rate on the farms as a result of the intervention as well as the most appropriate outcome measurement for the objectives of this study.

Other research has examined the impact of stewardship interventions within calf populations. Gomez et al. (2021) found reductions in antimicrobial use following the implementation of an algorithm-based system for identifying calves requiring antimicrobial therapy. This study was limited by the lack of a control group, in addition to its reliance on treatment records for estimations of antimicrobial use. Reliance on farm records underestimates AMU, and health records within calf productions systems are particularly problematic [17,18].

Cheng et al. (2022) found an overall treatment incidence (used daily dose) of 28.7 doses per 100 calf-days. Generally, there are few reports of quantified antimicrobial use in North America; however, antimicrobial use estimates are typically higher than those of other production systems due the inherent susceptibility of calves to bacterial infections. This is the only study that quantified AMU among veal calves in the United States, but estimates from European countries ranged as either higher in Belgium (38.7 per 100 calf-days), or lower in France [19]. Notably, more recent data in veal calves from Belgium suggest antimicrobial use has decreased [20].

### 4.4. Limitations

Random allocation of study subjects is a hallmark of experimental study designs [21]; however, random allocation of the study subjects (i.e., farms) was not possible due to a limited number of farms within driving distance from the university, and the requirement for the intervention farms’ willingness to receive the training materials. Confounding and other inadvertent biases are more difficult to eliminate for these types of studies; however, this is a limitation likely to be true of most educational interventions applied at the farm level. These studies are nonetheless important to provide evidence of effectiveness for the targeted outcome (i.e., reduced antimicrobial use). Additionally, the enrolled herds used written rather than digital record keeping systems, and the producers often did not record all treatments or disease-specific reasons for antimicrobial treatment. Therefore, differences in disease incidence between intervention and control herds could not be incorporated into the study. Although the farms were similar in many respects, it is possible that control herds had a coincidentally higher disease incidence, and that this confounded the results of the study. Future work should additionally include baseline data from the farms as well as measures of disease incidence, in order to reduce the risk of inadvertent confounding due to a small sample size; however, enrollment was limited by the number of farms within a drivable distance of the university. Certainly, larger studies that characterize the sources of between-farm variations in AMU are warranted. Regardless, this study supports that structured calf producer training can change treatment behaviors and result in important reductions in antimicrobial use. 

Antimicrobial stewardship should encompass all methods for reducing the quantity of medically important antimicrobials used on farms, while maintaining animal health and welfare and economic and social sustainability of the production system. The quantity of antimicrobials used for treatment is a product of disease incidence and selectivity, and therefore, AMS programs should include prevention of disease and more selective approaches for identifying animals that require antimicrobial therapy. Disease prevention in any production system is challenging, and particularly challenging in a veal production system where producers receive very young calves that are inherently susceptible to bacterial diseases, and have already experienced transport stressors and exposure to high-risk environments (e.g., livestock markets). The stewardship intervention reported here focused primarily on accurate case identification, but future AMS programs should more holistically include disease prevention. Additionally, future research should address methods for the efficient delivery of training materials, and endeavor to strengthen connections between veterinarians and farm personnel.

## 5. Conclusions

Calf producer-focused antimicrobial stewardship training resulted in improved knowledge, changed treatment behaviors and resulted in a 50% reduction in the quantity of individually administered antimicrobials. Additional efforts towards training farm personnel on essential competencies should be encouraged as a component of a holistic approach towards antimicrobial stewardship on farms.

## Figures and Tables

**Table 1:** Learning objectives for three modules of the didactic portion of an antimicrobial stewardship training program directed towards veal calf producers in Ohio.

Module	Learning Objectives
Antibiotics and Antibiotic Resistance	Describe the key differences between bacteria and viruses Differentiate between common uses for antibiotics, vaccines and other supportive therapies Define antibiotic resistance, and how it impacts animal and human health Describe why antibiotic stewardship programs are important
Calf Health Assessments	5. Identify sickness behaviors and calves that require further evaluation 6. Conduct a clinical evaluation 7. Categorize normal and abnormal clinical signs of disease 8. Describe scenarios when antibiotics, anti-inflammatories or fluids would improve the health outcomes
Decision Tree Protocols	9. Explain why treatment protocols are important 10. Describe the components of treatment protocols 11. Use treatment protocols to select appropriate individual calf treatment strategies 12. Distinguish between scenarios when treatment may or may not be needed

**Table 2:** Total antimicrobial use at individual and group levels in eight U.S. veal calf farms (2019–2020) during four rearing periods (entire grow, day 1–21, day 22–63 and day ≥64), expressed as the number of daily doses treated per 100 calf-days (TI100). Personnel on the intervention farms received training focused on antimicrobial stewardship and the accurate identification of cases.

	Study Group †	
Rearing Periods	Intervention (Mean TI100 ^‡^ ± SD ^§^)	Control (Mean TI100 ^‡^ ± SD ^§^)
Group treatments		
Entire period	9.48 ± 3.42	13.2 ± 5.06
Day 1–21	80.6 ± 40.8	64.0 ± 24.3
Day 22–63	21.5 ± 9.87	41.8 ± 20.5
Day ≥ 64	2.65 ± 0.87	4.70 ± 4.35
Individual treatments		
Entire period	12.8 ± 6.71	24.0 ± 6.17
Day 1–21	101.2 ± 75.1	83.8 ± 39.5
Day 22–63	29.6 ± 8.90	49.8 ± 16.1
Day ≥ 64	3.68 ± 2.63	7.76 ± 5.21

^†^ Four intervention and four control farms. ^‡^  $\mathrm{TI}100=\frac{\mathrm{total}\;\mathrm{mass}\;\mathrm{of}\;\mathrm{used}\;\mathrm{antimicrobial}\;(\mathrm{mg})}{\mathrm{Used}\;\mathrm{dose}\;(\mathrm{mg/kg})\;\times \;\mathrm{calf}\;\mathrm{weight}\;(\mathrm{kg})\;\times \;\mathrm{calf-days}\;\mathrm{at}\;\mathrm{risk}}\;\times 100.$ ^§^ SD = standard deviation.

**Table 3:** Group and individual antimicrobial use throughout the entire rearing periods in eight U.S. veal calf farms (2019–2020), expressed as the number of daily doses treated per 100 calf-days (TI100).

	Study Group †	
Antimicrobial Agents	Intervention (Mean TI100 ^‡^ ± SD ^§^)	Control (Mean TI100 ^‡^ ± SD ^§^)
Group treatments		
Amoxicillin	1.16 ± 0.40	1.21 ± 0.50
Chlortetracycline HCl	5.52 ± 2.05	5.01 ± 3.12
Lincomycin–oral ^¶^	0.98	1.69
Neomycin	1.00 ± 0.93	3.50 ± 3.25
Penicillin G potassium	1.04 ± 0.86	2.71 ± 1.75
Sulfamethoxazole + Trimethoprim	0.73 ± 0.30	1.72 ± 0.97
Tetracycline hydrochloride ^¶^	1.01	0.26
Individual treatments		
Amoxicillin	2.35 ± 0.59	2.68 ± 0.82
Ceftiofur sodium	0.64 ± 0.37	0.23 ± 0.21
Chlortetracycline HCl	7.06 ± 7.20	10.12 ± 2.86
Florfenicol	0.16 ± 0.06	0.17 ± 0.04
Lincomycin–parenteral	1.03 ± 0.46	2.87 ± 4.25
Lincomycin–oral ^¶^	0.03	1.65
Neomycin	1.09 ± 0.92	1.21 ± 0.64
Penicillin G potassium	0.90 ± 0.28	2.79 ± 1.12
Penicillin G procaine	1.93 ± 0.90	2.37 ± 0.97
Spectinomycin	0.14 ± 0.16	0.02 ± 0.00
Sulfamethoxazole and trimethoprim	0.80 ± 0.56	1.13 ± 1.05
Tetracycline hydrochloride ^¶^	1.69	-
Tildipirosin	0.07 ± 0.05	0.12 ± 0.12
Tulathromycin	0.17 ± 0.14	0.22
Tylosin	0.11 ± 0.12	0.09 ± 0.06

^†^ Four intervened and four control farms. ^‡^ $\mathrm{TI}100=\frac{\mathrm{total}\;\mathrm{mass}\;\mathrm{of}\;\mathrm{used}\;\mathrm{antimicrobial}\;(\mathrm{mg})}{\mathrm{Used}\;\mathrm{dose}\;(\mathrm{mg}/\mathrm{kg})\;\times \;\mathrm{calf}\;\mathrm{weight}\;(\mathrm{kg})\;\times \;\mathrm{calf}-\mathrm{days}\;\mathrm{at}\;\mathrm{risk}}\;\times 100.$ ^§^ SD = standard deviation. ^¶^ Oral lincomycin was administered for group treatment in Farms 7 and 8, and individual treatment in Farms 1 and 8. Tetracycline hydrochloride was administered for group treatment in Farms 1 and 8, and for individual treatment in Farm 1.

## Data Availability

Not applicable.
